# A Rare Case of Partial Small Bowel Obstruction Secondary to Intestinal Myeloid Sarcoma

**DOI:** 10.7759/cureus.52956

**Published:** 2024-01-25

**Authors:** Paige Barnard, Omar Mouline

**Affiliations:** 1 General Surgery, Cairns Hospital, Cairns, AUS; 2 General Surgery, Cairns Hospital/James Cook University, Cairns, AUS

**Keywords:** chemotherapy, surgery, acute myeloid leukaemia, myeloid sarcoma, small bowel obstruction

## Abstract

This case report details a rare case of small bowel myeloid sarcoma (MS) in an otherwise fit and well 49-year-old male presenting initially with vague obstructive symptoms and weight loss. The patient ultimately required an operation for a small bowel obstruction where a laparotomy and small bowel resection were performed due to three cicatrising completely obstructing lesions in the mid-jejunum. Fewer than 1% of patients with acute myeloid leukaemia (AML) present with MS as an initial diagnosis, and only 6.5% of these are intestinal in origin. This report adds to the current body of literature on this rare condition, emphasises the diagnostic challenges resulting in delays to diagnosis, and discusses the crucial role of early and accurate identification for optimal treatment and prognosis. Surgery may be warranted in patients with complications such as obstruction; however, systemic chemotherapy tailored to AML is the primary therapeutic approach for MS patients.

## Introduction

Myeloid sarcoma (MS) is a rare tumour that presents as an isolated extramedullary tissue mass form of acute myeloid leukaemia (AML) or, less commonly, a myeloproliferative disorder. It can occur concurrently, at relapse, or as a precursor to AML [1]. Due to the rarity of this disorder, most of the relevant literature comprises case reports. This report aims to add to the current literature body and highlight the issue of mis- or delayed diagnosis in this patient group.

Fewer than 1% of patients with AML present with MS as an initial diagnosis [2,3]. Potential sites of MS include soft tissues, bone, periosteum, lymph nodes, mediastinum, gastrointestinal tract, uterus, or ovaries [4]. In non-leukaemic patients diagnosed with MS, the average timeframe of progression to AML without chemotherapy is six months [5]. This case focuses on a presentation of small bowel MS, a rare oncological presentation with only 6.5% of MS cases presenting in the gut [6].

## Case presentation

This case presentation discusses a 49-year-old male who presented with progressive obstructive gastrointestinal symptoms and was diagnosed with MS after a small bowel resection. The patient initially presented to the emergency department complaining of eight weeks of worsening cramping central abdominal pain associated with anorexia and altered bowel habits (primarily loose). He had lost approximately 6-7 kg over this period and was feeling lethargic. He was otherwise fit and well with no known medical or surgical history.

**Figure 1:**
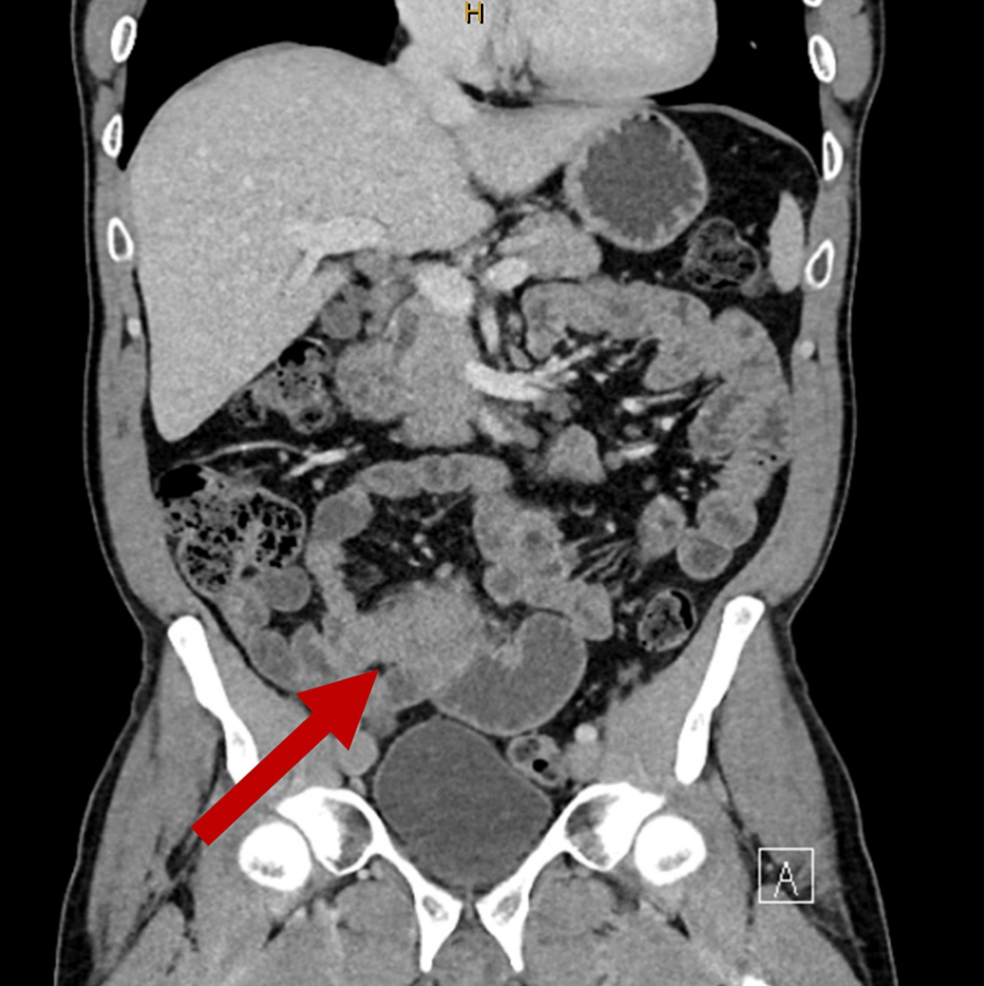
CT of the abdomen and pelvis with the red arrow demonstrating soft tissue mass extending into the mesentery with segmental thickening and high-grade stenosis of the mid and distal ileum, along with associated mesenteric lymphadenopathy and partial small bowel obstruction.

The patient underwent a CT scan which showed segmental thickening and high-grade stenosis of the mid and distal ileum, a soft tissue mass extending into the mesentery with associated mesenteric lymphadenopathy, and partial small bowel obstruction (Figure 1). The working differentials for the surgical team at this time were small bowel primary malignancy (i.e. neuroendocrine tumour or adenocarcinoma, lymphoma, and inflammatory bowel disease (IBD)). Given the segmental CT lesions, diarrhoea, weight loss, and a faecal calprotectin of 4,700 μg/g (normal range 10-60 μg/g), IBD was suspected at this time. The patient was planned for a double-balloon endoscopy (DBE) to access the mid and distal ileum as standard endoscopy would not necessarily reach the lesion in question.

**Figure 2:**
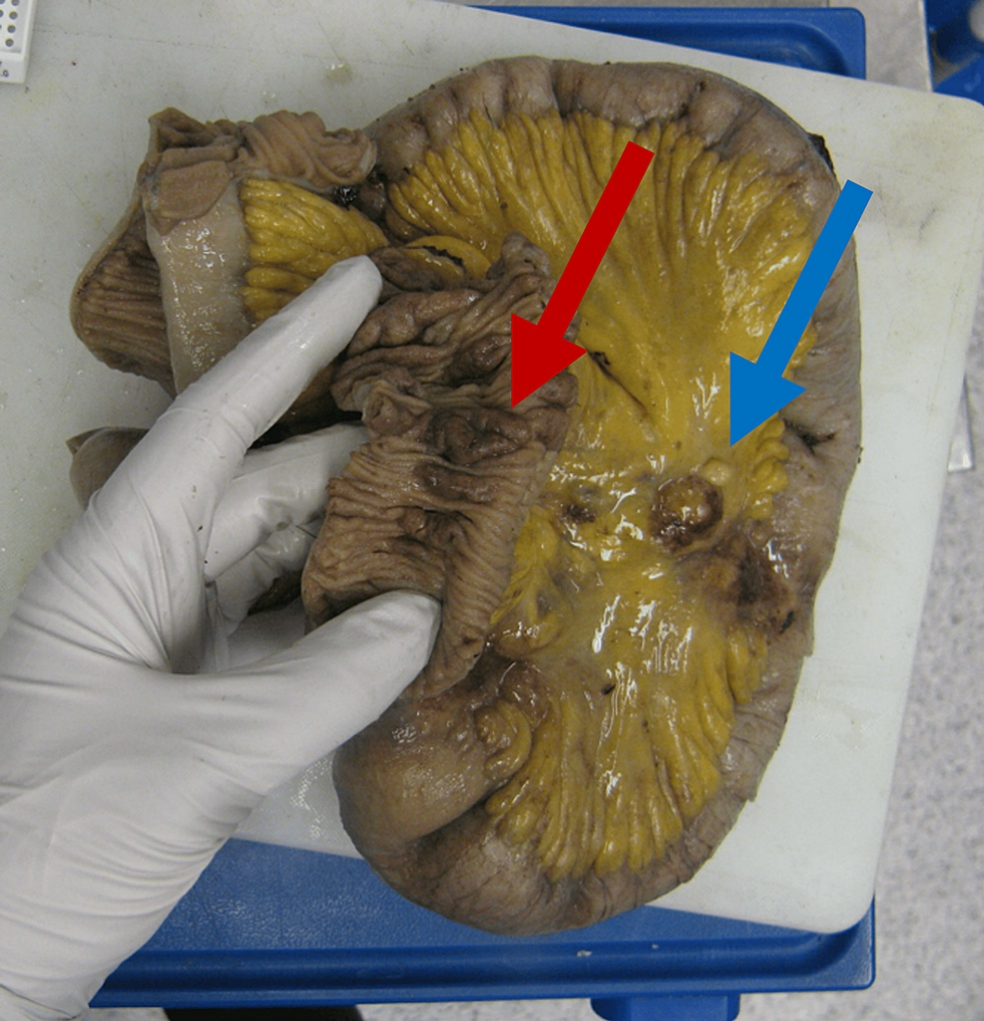
Resected small bowel specimen containing three stenotic circumferential small bowel lesions (one clearly on display: see the red arrow) and multiple enlarged mesenteric lymph nodes (see the blue arrow).

Unfortunately, DBE was unavailable at the time. Given the patient’s obstructive symptoms had resolved and they were tolerating a full diet, they were discharged from the hospital with a plan for follow-up in the clinic and an outpatient DBE. The patient was reviewed in the clinic a week later and seemed to have deconditioned further with ongoing weight loss, minimal oral intake, and intermittent obstructive symptoms. The patient was re-admitted to the hospital with a plan for an endoscopy and possible semi-emergent diagnostic laparoscopy, dependent on findings. However, on admission to the hospital, the patient was found to be clinically completely obstructed and did not improve. Given this, the patient ultimately proceeded to an operation where a diagnostic laparoscopy was converted to an open laparotomy due to concern for malignancy and the need for a small bowel resection. Findings included a dilated small bowel leading to three cicatrising completely obstructing lesions in the mid-jejunum with collapsed small bowel distally and multiple enlarged lymph nodes noted within the mesentery. There was no evidence of peritoneal or solid organ metastatic disease. A total of approximately 2 m of small bowel was resected to include the lesions described above, and a hand-sewn end-to-end anastomosis was formed (Figure 2).

**Figure 3:**
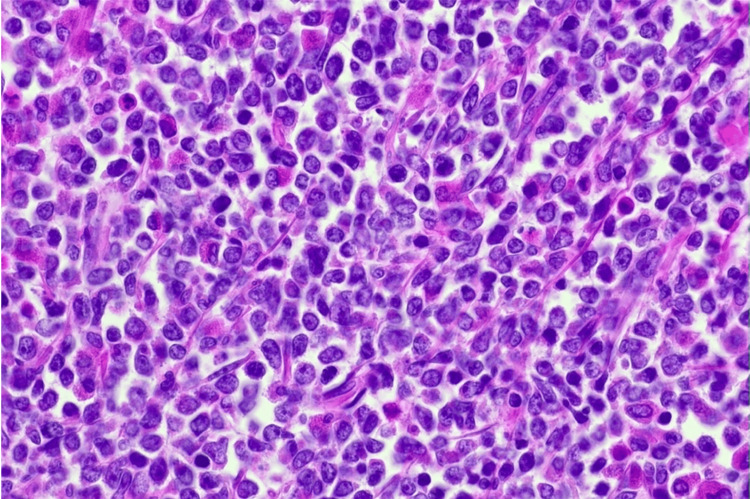
Histopathology slide demonstrating the key features of MS; immature medium-sized myeloid cells with finely granular eosinophilic cytoplasm and typical cleaved/reniform nuclei with fine blastic chromatin and small nucleoli.

Histopathology from the operation returned as MS. The sections of the small bowel and lymph nodes showed similar features. The small bowel showed effacement of normal architecture along with mass forming diffuse infiltrate of neoplastic cells with cleaved nuclei, high nucleus-to-cytoplasmic ratio, and dispersed chromatin (Figure 3). Many of the cells appeared plasmacytoid. Mitotic figures were readily identified. The cells were positive for CD45, CD117, CD68, MPO, and BCL2 but negative for CD3, CD20, CD10, CD23, CD5, cyclin D1, and CD138. Ki-67 showed a proliferation index of up to 80%. The lymph nodes also showed infiltration by similar cells. The overall features were reported to be in keeping with MS.

From here the haematology team was involved and an emergent bone marrow aspiration and trephine biopsy was arranged. The results showed no evidence of AML, with normocellular marrow, adequate trilineage haematopoiesis, no increase in blasts, and normal flow cytometry. A positron emission tomography (PET) CT scan showed pronounced nodal fluorodeoxyglucose avidity at several locations above and below the diaphragm, including the right supraclavicular fossa node, mediastinal pre-vascular node and right inguinal nodes. The patient underwent systemic treatment with chemotherapy (high-dose cytarabine and Idarubicin), followed by an allogeneic stem cell transplant. Six months later they had not developed AML. A post-treatment PET scan showed a complete response with no evidence of local or metastatic disease.

## Discussion

Small bowel tumours can be either primary or metastatic, with metastasis being more common. Cancers causing metastatic small bowel tumours include melanoma, lung cancer, and breast cancer. Primary small bowel tumours include adenocarcinomas, lymphomas, gastrointestinal stromal tumours, and sarcomas [7]. MS, typically an extramedullary manifestation of AML and less commonly associated with other myeloproliferative disorders, is an exceptionally rare occurrence, particularly in isolated or de novo cases [1]. While many patients with intestinal MS may be asymptomatic or present with vague non-specific symptoms, complications documented in the literature include haemorrhage, perforation, necrosis, obstruction, and intussusception [7].

Pre-histopathological diagnosis of primary small bowel MS can be extremely challenging due to the inherent rarity of the disease and the non-specific nature of the clinical presentation and radiological features. The misdiagnosis of MS presents a substantial challenge in clinical settings. However, timely diagnosis is key as the mainstay of treatment is systemic chemotherapy. Surgical resection, while sometimes necessary due to complications such as obstruction, shows no actual improvement in overall survival [5]. Reports suggest that up to half of patients with MS are initially misdiagnosed, often as having a primary or metastatic malignant tumour, particularly malignant lymphoma [7-17]. This mis- or delayed diagnosis has been reported to occur both at initial primary differential diagnosis and even post-biopsy or resection with misinterpretation of histopathology [9-11,17]. Our case highlights this diagnostic challenge.

Imaging findings are often inconsistent and non-specific and cannot be relied upon to differentiate intestinal MS. CT findings of MS involving the small bowel and colon manifest diversely, presenting as intraluminal or exophytic polypoid masses, bowel wall thickening, or a combination of these features. However, contrast enhancement patterns are variable, posing challenges in reliably differentiating MS from lymphoma, other neoplastic conditions, or IBD based solely on CT findings [7,8,11,17-19].

Early and accurate diagnosis of MS is imperative due to its impact on treatment regimens [1,9]. The prognosis of isolated MS is variable but better if diagnosed early [14]. Surgical resection is a common intervention for treating intestinal obstruction associated with MS, as observed in our case. However, it is crucial to emphasise that systemic chemotherapy tailored to AML is the primary therapeutic approach for MS patients [1]. Surgical intervention has not been proven to influence survival; however, it may be indicated for complications such as obstruction or perforation [7]. As in our case, the patient was deteriorating from an obstructive perspective, and surgery ultimately treated the obstruction and allowed for simultaneous diagnosis. While most general surgeons will not encounter this diagnosis frequently, it is important to raise clinical awareness of this rare condition so it can be considered as a potential differential diagnosis for small bowel tumours.

Systemic chemotherapy, especially AML-type induction chemotherapy, can delay the time to develop AML and prolong the survival period [14]. Studies have underscored the significance of early diagnosis, showing a significantly longer non-leukaemic interval after diagnosis in patients who received chemotherapy compared to those who did not [20]. A series of 74 cases of isolated granulocytic sarcoma (42 who received chemotherapy and 32 who did not) showed that all 32 of the patients who did not undergo systemic treatment ultimately progressed to AML, with 81% progressing within the first 11 months. Overall, 58% (24 patients) who received chemotherapy were disease-free for up to 11 months and 19% (8 patients) for up to two years [21].

Furthermore, there is evidence to support allogeneic hematopoietic stem cell transplantation (allo-HSCT) as first-line MS treatment following chemotherapy. Chevallier et al. conducted a retrospective multi-centre study including 99 patients (the largest series of isolated MS patients having undergone allo-HSCT). They reported a five-year overall survival and leukaemia-free survival of 48% and 36%, respectively; given the usually reported overall survival of less than two years, this suggests allo-HSCT is an effective treatment option for isolated MS [22].

## Conclusions

In summary, this report discusses the rare and unusual case of a 49-year-old male who required a laparotomy and small bowel resection for a bowel obstruction, secondary to MS. The patient subsequently received systemic chemotherapy and a bone marrow transplant. At this point, approximately six months post-surgery, the patient remains disease-free and well in the community. This case adds to the current body of literature on the rare presentation of small bowel MS. Common issues of delayed and difficult diagnosis are highlighted, emphasising the need for increased clinical awareness. Surgical resection, although not proven to impact survival, remains a sometimes necessary option for managing complications, but systemic chemotherapy tailored to AML remains the primary therapeutic approach for MS patients. There is also evidence to support allo-HSCT as first-line therapy for MS.
